# Supplementary material for: Machine learning in dentistry: a scoping review
Source: PLOS Digit Health. 2025 Jul 23;4(7):e0000940. doi: 10.1371/journal.pdig.0000940 (PMC12286321; doi:10.1371/journal.pdig.0000940)
Supplement: S1 Table — The search strategy is provided in the supporting information file labeled “S1_Table.pdf”. (PDF) [file pdig.0000940.s003.pdf]

S1 Table. Search strategy for PubMed.

(“machine learning”[MeSH Major Topic] OR “Electronic Health Records”[MeSH Terms] OR “Electronic Health Records”[Title] OR “informatics”[MeSH Terms] OR “informatics”[Title] OR “decision support systems, clinical”[MeSH Terms] OR “clinical decision support”[Title] OR (“machine”[Title] AND “learning”[Title]) OR “machine learning”[Title] OR “AI”[Title] OR “Artificial Intelligence”[Title] OR “artificially intelligent”[Title] OR “Artificial Intelligence”[MeSH Terms] OR “Algorithms”[MeSH Terms] OR “algorithm”[Title] OR “deep learning”[Title] OR “computer vision”[Title] OR “natural language processing”[Title] OR “neural network”[Title] OR “neural networks, computer”[MeSH Terms] OR “intelligent machine”[Title]) AND (“dentistry”[MeSH Major Topic] OR “dentistry”[Title] OR “dental”[Title] OR (“oral”[Title] AND “medicine”[Title]) OR “oral medicine”[Title] OR “oral medicine”[MeSH Terms] OR “stomatology”[Title] OR “evidence based dental practice”[Title]) AND 2018/01/01:2023/12/31[Date - Publication]
