# Supplementary material for: Machine learning in dentistry: a scoping review
Source: PLOS Digit Health. 2025 Jul 23;4(7):e0000940. doi: 10.1371/journal.pdig.0000940 (PMC12286321; doi:10.1371/journal.pdig.0000940)
Supplement: S2 Table — The evaluation criteria are provided in the supporting information file labeled “S2_Table.pdf”. (PDF) [file pdig.0000940.s004.pdf]

S2 Table. Evaluation criteria for included studies.

Criteria	Explanation	TRIPOD+AI domain	Data type / values
Identify clinical goal	The desired clinical outcome of the model, typically mentioned in the abstract or introduction.	Background, objectives, and outcomes	Boolean (Yes/No)
Dental specialty	The branch of dentistry addressed, based on standardized specialty definitions.	Study design and setting	Categorical (Oral and maxillofacial radiology, Oral and maxillofacial surgery, General dentistry, Prosthodontics, Orthodontics and dentofacial orthopedics, Periodontics, Oral and maxillofacial pathology, Endodontics, Pediatric dentistry, Oral medicine, Orofacial pain, Dental anesthesiology)
Type of model under investigation	The type of machine learning model studied.	Development of the prediction model	Categorical (Classification, Segmentation, Regression, Generative AI)
Type of machine learning Approach	The type of machine learning approach used.	Development of the prediction model	Categorical (Supervised ML, Unsupervised ML)
Define prediction outcome	Whether the model's predicted outcome was clearly defined in the paper, e.g., diagnosis or prognosis.	Background, objectives, and outcomes	Boolean (Yes/No)
Retrospective vs Prospective	Whether the study used retrospective (past data) or prospective (future-oriented) data.	Study design and setting	Categorical (Retrospective, Prospective, Both)
Comparison with reference standard models	Whether the model was compared with existing models, clinical settings, or dentist-derived metrics.	Model performance, calibration, and validation	Boolean (Yes/No)
Inclusion/exclusion criteria of participants	How participants or datasets were selected, based on criteria stated in the methods section.	Study design, participants, and data sources	Boolean (Yes/No)
Define predictor variables	Variables used to predict the outcome, described in the paper, e.g., imaging modalities or structured data.	Predictors and model input	Boolean (Yes/No)
Data preprocessing/cleaning/transformation	Steps for handling data after acquisition but before modeling, including normalization, filtering, etc.	Data preparation and preprocessing	Boolean (Yes/No)
Outliers removed?	Whether outliers were removed or handled explicitly in the study.	Data preparation and preprocessing	Boolean (Yes/No)
Method for selection of outliers	The method used to identify outliers, e.g., statistical methods or imaging noise detection.	Data preparation and preprocessing	Boolean (Yes/No)
Were poor quality/missing data handled?	Whether missing or poor-quality data were addressed, e.g., through imputation or exclusion.	Data preparation and preprocessing	Boolean (Yes/No)
Method for handling poor/missing data	Specific methods for dealing with missing or poor data, e.g., statistical imputation or clinical judgment.	Data preparation and preprocessing	Boolean (Yes/No)
Validation strategies	Type of validation applied, e.g., k-fold cross-validation, internal vs external validation, bootstrap.	Model validation	Boolean (Yes/No)
Justify decision for using chosen model	Whether the paper provided reasons for choosing the particular model, even if based on prior studies.	Rationale for model choice	Boolean (Yes/No)
Methods detailed enough to reproduce?	Whether the methodology provided sufficient detail for reproducibility, e.g., GitHub links or appendices.	Model reproducibility and reporting	Boolean (Yes/No)
Dataset publicly available?	Whether the dataset used in the study is publicly accessible.	Reproducibility and data sharing	Boolean (Yes/No)

Continued on next page

S2 Table continued from previous page

**Criteria**	**Explanation**	**TRIPOD+AI domain**	**Data type / values**
Report performance of model: Discrimination	Metrics like AUROC or precision/recall for classification tasks, or RMSE/R^2 for regression tasks.	Model performance and validation	Boolean (Yes/No)
Report performance of model: Calibration	Metrics that show the accuracy of predicted probabilities, e.g., Brier score, calibration plots.	Model Performance and Calibration	Boolean (Yes/No)
Report performance of model: Clinically relevant	Metrics like PPV and NPV that have practical clinical implications for decision-making.	Model performance and clinical relevance	Boolean (Yes/No)
Report parameter estimates and confidence intervals	Details of parameter estimates, confidence intervals, or hyperparameters of the model.	Model performance and hyperparameters	Boolean (Yes/No)
Interpretability of model (e.g., SHAP)	Whether the model's decision-making was explained using interpretability tools like SHAP or saliency maps.	Model interpretation and explainability	Boolean (Yes/No)
Bias and fairness	Whether the paper acknowledged or addressed generalizability, bias, or equity concerns.	Bias, fairness, and generalizability	Boolean (Yes/No)

Notes: The table summarizes the evaluation criteria applied to the included studies in the scoping review of machine learning in dentistry.
