# Supplementary material for: Machine learning in dentistry: a scoping review
Source: PLOS Digit Health. 2025 Jul 23;4(7):e0000940. doi: 10.1371/journal.pdig.0000940 (PMC12286321; doi:10.1371/journal.pdig.0000940)
Supplement: Attachment — Submitted filename: Response to Reviewers.docx [file pdig.0000940.s005.docx]

Dear Editor,

Thank you for reviewing our manuscript. Your comments and suggestions have greatly improved our work.

Below is our detailed response to each reviewer's comment. We have addressed all concerns and made necessary revisions. Each comment is listed in bold, followed by our response and any changes.

We hope the revised manuscript meets the standards of PLOS Digital Health.

Regards,

**Reviewer #1: This scoping review provides a much-needed overview of the landscape of machine learning in dentistry. It sheds light on important shortcomings in current research (notably the lack of calibration reporting, bias considerations, and open data). The paper is generally well-organized and informative. I offer the following constructive suggestions to further enhance the clarity and impact of the review:** 
 
**• The Introduction could be made more concise, for example, the sentence “Dentistry... is ready to gain a lot from these advancements” could be rephrased in a more scholarly tone. Additionally, it’s better to avoid repetition – the phrase “to the best of our knowledge” appears twice in proximity.** 

Thank you for the suggestions, we have incorporated them in the Introduction section. (Page 3)

**• The Authors mention that previous reviews have explored ML in dentistry but that this work is unique in emphasizing explainability of metrics, calibration, and bias (which is an excellent positioning). It would strengthen the introduction to briefly cite those prior reviews and explicitly state how this review differs from them.** 

Thank you for this suggestion, we have added the relevant examples for the reviews. (Page 3)

**• The methodology is generally well-detailed and rigorous. One concern is the restriction to PubMed-indexed articles. Considering the interdisciplinary nature of ML, relevant studies might appear in computer science venues or other databases. In the manuscript’s Limitations, The authors acknowledge that limiting to PubMed and English could exclude some studies. To preempt readers’ concerns, it would help to briefly justify this in the Methods as well – for example, stating that PubMed was chosen for its coverage of biomedical journals.**

Thank you, we added the justification. (Page 4) 
 
**• The authors should perhaps specify whether the multi-investigator reviewing process applied to full-text screening as well (it’s currently implied).**

Thank you for this suggestion, we have added details in the Data collection process subsection about the two-stage review process and how multiple investigators were involved at each step. (Page 4) 
 
**• One suggestion is to clarify in the Methods how the TRIPOD+AI rubric was applied. TRIPOD+AI is a reporting guideline; did the authors score each study on certain checklist items, or simply used it as an informal guide to decide which information to record (like calibration, interpretability, bias)?**

Thank you for this suggestion, we have added details in the Data collection process subsection about the TRIPOD+ AI guideline being applied to Stage two of the review process. The supplementary table 2 lists each of the elements being recorded. We have updated the table to include another column named “Data Type / Values” where we further succinctly write how we recorded the data. (Page 4) 
 
**• One area the authors might want to expand on is model validation approaches. Many readers will wonder: how many studies performed external validation or multi-center evaluation versus those that only did internal validation?** 

Thank you for this suggestion. This is something which did come to our mind while writing the manuscript but since we recorded data as Yes/No for this element- it would not be possible in the current scope of work to add it. But in our future work, we do plan to keep this in mind.

**• Table 1 succinctly summarizes key performance metric reporting frequencies, which is a great addition. Depending on journal space, the authors might consider an additional figure or table to enrich the results. For example, a bar chart of the number of studies per dental specialty or per year could visually illustrate trends.**

Thank you for this suggestion. A stacked column chart depicting number of studies for different dental specialties per year has been incorporated in Figure 2. (Page 8)

 
**• To further enrich the discussion, the authors can consider adding concrete examples or referencing known techniques. For instance, when discussing bias and fairness, they can mention subgroup analysis and fairness-aware algorithms as remedies– citing a relevant study or framework (perhaps outside dentistry) that successfully applied such techniques, to illustrate what future dental ML studies could do.**

Thank you, we have added an example in the “Equity, bias, and fairness considerations” subsection referencing how subgroup analysis can be used to mitigate this. We have also added more details on how future dental ML studies can address this in the “Limitations and future directions” subsection. (Pages 9,10)

 
**• Since this is a scoping review, it would be helpful to explicitly state what the implications are for different stakeholders. The authors can consider mentioning implications for practitioners or educators.**

Thank you, we have added a subsection “Implications for Stakeholders” under the Discussion section and described the implications for the different stakeholders. (Page 11)

**• One additional future direction to mention could be the development or adoption of community data repositories or benchmarks for dental AI. Given that small number of studies shared their data publicly, the field would benefit from common datasets.**

Thank you for the suggestion, we have included this in the “Limitations and future directions” and the “Implications for Stakeholders” subsections. (Pages 10,11)

**• Minor points: Overall, the manuscript is well-written. There are a few minor typographical and grammatical errors to correct for a polished final version. For example, “knolwedge” should be “knowledge”, and “simulatenously” should be “simultaneously”.**

Thank you so much! We have made those changes and checked again for any such mistakes.

**Reviewer #2: This manuscript addresses an important and rapidly evolving topic—the use of machine learning (ML) in dentistry—and commendably covers a wide breadth of studies. While the review is comprehensive in scope, incorporating the following refinements could further enhance clarity, transparency, and methodological rigor:** 
**1. While scoping reviews can serve exploratory purposes, transparency and reproducibility would be further strengthened if a protocol is registered or published a priori (e.g., on Open Science Framework (OSF) or in journals that accept scoping review protocols). Just as a note for future scoping reviews: PROSPERO does not accept scoping review protocols.**

Thank you so much for this feedback. We have updated the database name to “Open Science Framework” from “PROSPERO” in the “Materials and methods” section. We will keep this in mind for future reviews. (Page 3)

**2. Although PRISMA-ScR was used for reporting, it is unclear what methodological framework guided the conduct of the review (e.g., JBI methodology guidance for scoping reviews, Arksey and O'Malley, or its updated versions). Clarifying this could provide readers with greater insight into the review process.**

Thank you for this suggestion! We have updated the “Materials and methods” section to include this. (Page 3)

**3. Including the study types (study design) in the eligibility criteria could assist readers in understanding the weight and diversity of the evidence reviewed. Additionally, describing the study types in the "Characteristics of Included Studies" section would provide a clearer overview of the included literature.**

Thank you, we have added details in the “Inclusion criteria” subsection about the Study type. Since we did not capture more information about the types of studies, creating a new "Characteristics of Included Studies" section with those details would be out of our current scope but will keep in mind for future research. (Page 5)

**4. Is there a specific rationale for selecting the time frame 2018 to 2023? If so, it would be helpful to mention this explicitly in the eligibility criteria.**

Thank you for the suggestion. We captured these years to show the trends in this field over a larger time period and since there has been a huge surge in interest in this topic over the last couple of years we selected this range. We have added those details in the “Inclusion Criteria” subsubsection. (Page 5)

**5. Since the PICO framework is used, consider integrating it directly into the inclusion criteria section. This could streamline the methodology and avoid redundancy.**

Thank you for the suggestion. We have revised and added the PICO elements under the “Inclusion Criteria” subsubsection. (Page 5)

**6. The PRISMA flow diagram could be further improved by including details such as the number of duplicates removed, specific reasons for article exclusion, and information at each screening stage.**

Thank you for the suggestion. We have revised and added the screening stages and studies removed under appropriate reason in Fig 2. (Page 8)

**7. The PRISMA-ScR checklist item “Synthesis of Results” is listed as reported on Page 4, but there is no clear description of how results will be synthesized on that page. This section could be a suitable place to describe the planned analyses, such as subgroup analysis or other methods of result synthesis.**

Thank you for this suggestion. We have added details under the subsection “Synthesis of results” under the “Materials and methods” section. (Page 6)

**8. It may be helpful to provide a comprehensive description of the characteristics of included studies (e.g., study design, geographical distribution) and use a thematic approach to present the findings in a more concise and organized manner, which could improve readability.**

Thank you for this pertinent suggestion. We have added a stacked column chart depicting the number of studies for different dental specialties per year in Figure 2. (Page 8)

We have also incorporated details in the “Inclusion criteria” subsubsection about the Study type. Since we did not capture more information about the types of studies, creating a new "Characteristics of Included Studies" section with those details would be out of our current scope but will keep in mind for future research. (Page 5)

**9. You highlighted the need to address equity and bias; however, providing specific examples from the reviewed literature could better demonstrate potential real-world implications or consequences of unaddressed biases.**

Thank you for the suggestion! We have incorporated an example in the “Equity, bias, and fairness considerations” subsection. (Page 8)

10**. Including practical recommendations for how dental practitioners can critically evaluate ML models before integrating them into clinical practice could further enhance the applicability of your findings.**

Thank you for the suggestion! We have incorporated recommendations for integration of ML into clinical practice in the “Implications for Stakeholders” section. (Page 11)

**11. While you acknowledged the limitation regarding the exclusive use of PubMed, it may be worth explicitly recommending that future reviews incorporate additional databases (e.g., Embase, Scopus) to ensure a more comprehensive literature search**.

Thank you for the suggestion, we have added need for comprehensive search in future reviews using additional databases in the “Limitations and future directions” subsection. (Page 10)

**12. Minor Comments and Corrections:** 
**• Grammar and Proofreading: Page 3, line 24: typo correction — "knolwedge" → "knowledge".** 
**• Percentage Reporting: Ensure consistency in reporting percentages (e.g., use either 60% or 59.8% consistently).**

Thank you so much! We have made those changes and checked again for any such mistakes.

**• Figures: Consider including more graphical summaries (e.g., visual representations of dental specialties and ML tasks) to facilitate quick interpretation for readers.**

Thank you for this suggestion. A stacked column chart depicting number of studies for different dental specialties per year has been incorporated in Figure 2. (Page 8)

**Reviewer #3: The authors perform a scoping review of the dentistry literature to assess ML models in dentistry from 2018 to 2023. They follow the PICO framework and using PRISMA-ScR.** 
 
**The overall quality of the review is high with careful attention paid to critical details regarding potential interpretability and downstream translation of these models. The emphasis on calibration and bias of the models is particularly important given the impact these elements will have on downstream translation. The search strategy only included PubMed and did not include IEEE Xplore for more technical type papers which may have offered greater methodological detail in modeling strategies. The authors should note the use of only one database in their limitations.**

Thank you for the suggestion, we have added need for comprehensive search in future reviews using additional databases in the “Limitations and future directions” subsection. (Page 10)

 
**It would be helpful also to understand how many studies were in each type of application, for example, computer vision vs. tabular analysis to achieve its relevant tasks of classification, segmentation, etc.**

 
**Minor:** 
**In the manuscript there is use of informal English such as "can't" rather than cannot and "couldn't" rather than could not. Would defer to the publisher regarding appropriate use of contractions.**

Thank you for your suggestion! We have made the changes.

**Reviewer #4: General Assessment:** 
**The manuscript explores a timely and relevant topic with well-defined objectives and a structured methodology, appropriate for a scoping review. However, several critical issues must be addressed before it can be considered for publication.** 
 
**Major Concerns:** 
 
**Language and Style:** 
**The manuscript requires thorough language editing. Grammatical errors and awkward phrasing affect clarity. Redundancies (e.g., “To the best of our knowledge” appearing twice) and placeholders (e.g., "REFERENCE") should be corrected.**

Thank you! We have made the required changes.

 
**Methods:** 
**The definition of machine learning is unclear. For example, exclusion criterion #1 may confuse traditional statistical methods with ML. The description of evaluation criteria lacks details on scoring, instruments used, and reviewer agreement resolution. The absence of PROSPERO registration needs clearer justification.**

Thank you! We have justified the same in the “Material and Methods” section. (Page 3)

 
**Results Interpretation:** 
**Although percentages are presented clearly, the discussion is often superficial. Important findings, like the low reporting of calibration metrics, should be connected to their clinical relevance. Repetitive phrases should be consolidated, and vague language replaced with more precise analysis.**

Thank you for your suggestion! We have made the changes in the “Model performance metrics” subsection. (Page 9)

**Bias, Equity, and Reproducibility: The treatment of fairness and bias is shallow.**

**Mentioning that 25% of studies addressed bias is not enough—examples and tools (e.g., AIF360, Fairlearn) should be discussed. Similarly, the low data sharing rate (11.72%) demands stronger commentary on its impact on reproducibility and open science.**

Thank you for the suggestion. The same has been added under the “Limitations and Future Directions section.” (Pages 10,11) 
 
**Discussion and Conclusion:** 
**The discussion is generally well-structured, with relevant ethical and regulatory context. However, claims about unsupervised learning trends contradict the data and should be revised. The discussion on interpretability needs concrete examples. Limitations should be more deeply analyzed, especially regarding the exclusive use of PubMed. The conclusion lacks a strong final message and should include 2–3 practical recommendations for future research.**

Thank you for the suggestion! We have incorporated the changes in the “Discussion” section. (Pages 9, 10, 11)
